# Functional differentiation related to decomposing complex carbohydrates of intestinal microbes between two wild zokor species based on 16SrRNA sequences

**DOI:** 10.1186/s12917-021-02911-z

**Published:** 2021-06-11

**Authors:** Yao Zou, Nannan Liang, Xuxin Zhang, Chongxuan Han, Xiaoning Nan

**Affiliations:** Key Laboratory of National Forestry and Grassland Administration on Management of Western Forest Bio-Disaster, Northwest Agriculture and Forestry University, Yangling, 712100 China

**Keywords:** Zokor, Intestinal microbes, Fermentation, Cellulose, Adaptation

## Abstract

**Background:**

The intestinal microbes in mammals play a key role in host metabolism and adaptation. As a subterranean rodent, zokor digs tunnels for foraging and mating. These digging activities of zokors increase the energy expenditure relative to their aboveground counterparts. However, relatively little is known regarding intestinal microbes of zokor and how they make full use of limited food resources underground for high energy requirements.

**Results:**

*Eospalax cansus* and *Eospalax rothschildi* had distinct intestinal microbes. Although the composition of intestinal microbes is similar in two species, the proportion of bacterium are distinctly different between them. At phylum level, 11 phyla were shared between two species. Firmicutes and Bacteroidota were two dominant microbes in both of two species, while *Eospalax cansus* have a significantly high proportion of Firmicutes/Bacteroidota than that of *Eospalax rothschildi*. At genus level, *norank_f_Muribaculaceae* were dominant microbes in both of two zokor species. The relative abundance of 12 genera were significantly different between two species. Some bacterium including *unclassified_f__Lachnospiraceae, Lachnospiraceae_NK4A136_group, Ruminococcus* and *Eubacterium_siraeum_group* associated with cellulose degradation were significantly enriched in *Eospalax cansus*. Although alpha diversity was with no significant differences between *Eospalax cansus* and *Eospalax rothschildi*, the intestinal microbes between them are significant distinct in PCoA analysis. We have found that trapping location affected the alpha diversity values, while sex and body measurements had no effect on alpha diversity values. PICRUSt metagenome predictions revealed significant enrichment of microbial genes involved in carbohydrate metabolism in *Eospalax cansus* rather than *Eospalax rothschildi*.

**Conclusions:**

Our results demonstrate that *Eospalax cansus* harbor a stronger ability of fermentation for dietary plants than *Eospalax rothschildi*. The stronger ability of fermentation and degradation of cellulose of intestinal microbes of *Eospalax cansus* may be a long-time adaptation to limited food resources underground.

**Supplementary Information:**

The online version contains supplementary material available at 10.1186/s12917-021-02911-z.

## Background

Zokor is one of subterranean rodents endemic to east Asia [1]. It gnaw the roots of plants for food and could cause large-scale disasters to woodland and meadow [2]. Due to its special underground lifestyle, it is difficult to capture zokor so that the study of zokor is limited. More attentions are paid to the classification and phylogeography of zokor [3–6], while the researches on physiology and ecology of zokor are scarce. As a subterranean rodent, zokor has harbored many adaptive characteristics during long-time process of evolution, such as vestigial eyes, strong forelimbs [7, 8] and developed cecum [9, 10]. However, there are interspecies differences in these adaptive characteristic due to the differences of genetic background and living conditions. It is of great significance to compare and study the adaptive characteristics of different species of zokor.

The genomes of intestinal microbes in mammals contain more than 100 times as many genes as the host genomes [11]. The diversity of intestinal microbes plays an important role in maintaining the intestinal microflora and body balance. The composition and metabolites of intestinal microbes are closely related to the health status of host. Intestinal microbes have many physiological functions, they not only affect the digestion and absorption of food, but also regulate nutrition and immune response of host, and help the host to obtain energy from food [12]. One of the core functions is to help the host to decompose the complex polysaccharides which escaped from digestion of foregut [13]. As a phytophagous mammal, developed cecum of zokor indicates the morphological adaptation of digestive organs [10]. It is suggested that zokor has strong fermentation ability for complex polysaccharides because the expansion of cecum can extend the residence time of food in gut and thus improve the utilization of food [9]. However, there are little researches on intestinal microbes of zokor, and how limited food resources underground are fully utilized by intestinal microbes of zokor is still unknown. It is worth investigating the intestinal microbes of zokor and comparing the differences between different zokor species.

Intestinal microbes and hosts together formed relatively stable mutualism in the long-time process of evolution. Firmicutes, Bacteroidota and Actinobacteria are three dominant microbes of mammals [14]. Intestinal microbes of mammal are mainly influenced by host and environment factors [14, 15]. Host factors include genetic background, age, gender and the health status of host, while environment factors include food, season, environment microbes and geographical location. It was found that the season had significant impact on both community structure and diversity of cecal bacteria of *Eospalax cansus* (*E. cansus*). And this seasonal variation of zokor intestinal microbes was related to seasonal changes in food resource [16]. By feeding *E. cansus* on different types of plants, it was found that different food composition influence gut microbiota of zokor [17]. Zokors fed by woody plants have a higher proportion of Firmicutes/Bacteroidota and higher diversity compared to zokors fed by herbs. High proportion of Firmicutes/Bacteroidota of intestinal microbes of zokor was a response to the high-fiber food resources, and it would help zokor to obtain more energy from dietary plant.

Extant zokors (Myospalacinae) consist of two genera, *Eospalax* and *Myospalax* [4, 18]. Zokors in *Eospalax* are endemic species of China. Molecular evidences based on mitochondrial genome suggested that *E. cansus* and *Eospalax rothschildi* (*E. rothschildi*) are closely related in *Eospalax* [19]. However, the living conditions of habitats between these two species are distinct. *E. cansus* lives in the Loess Plateau area (34.68–41.60 °N, 100.55–107.94 °E) located in the northwest of China where the vegetation is sparse [20], and it often takes roots of woody plants as food because edible plant resources are limited. Differently, *E. rothschildi* lives in Qinba mountain area (31.13–32.60 °N, 107.55–113.06 °E) located in the south-central region of China, where the habitat is with relatively high annual mean temperature, plentiful rainfall, mixed evergreen and deciduous broad-leaved forest [21]. The edible types of plants for *E. rothschildi* are relatively abundant and various, including woody plants and herbaceous plants. The composition and structure of intestinal microbes of these two species and influencing factors seem to be unclear. This study investigated the composition and diversity of intestinal microbes of *E. cansus* and *E. rothschildi*, and compared the differences of intestinal microbes between these two species. Furthermore, the functions of intestinal microbes of *E. cansus* and *E. rothschildi* were predicted to reveal the functional differentiation of intestinal microbes between two zokor species. These findings would provide novel insights into the contributions of intestinal microbes to adaptive evolution of zokors and the process of species differentiation of Myospalacinae.

## Results

### Composition and relative abundance of intestinal microbes between E. cansus and *E. rothschildi*

After filtering out low-quality sequences, chloroplasts, chimeras and singletons, we obtained 2,138,122 valid sequences from 55 samples. To standardize sampling efforts across samples, each sample was rarefied to 22,494 sequences. With the increase of data size, the OTU-level rarefaction curves (Fig. S1) gradually reached stable values, indicating that the sequencing depth has reached the requirement. A total of 1796 OTUs, 12 phyla and 180 genera were detected in the gut microbiota of all zokors, and 1233 OTUs, 11 phyla and 133 genera were shared in two species, respectively (Fig. S2).

**Fig. 1:**
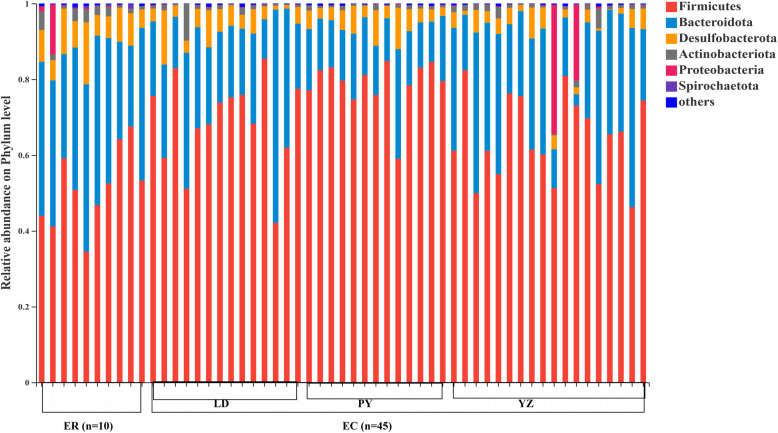
Mean relative abundances of bacteria at phylum level across all zokor samples (EC, *E. cansus*; ER, *E. rothschildi*)

At phylum level, the zokor gut bacteriome across all samples were dominated by Firmicutes and Bacteroidota, followed by Desulfobacterota, Actinobacteriota and Proteobacteria (> 1% relative abundance) (Fig. 1). We compared the difference of gut microbiota composition between *E. cansus* and *E. rothschildi*. *E. cansus* has more microbes than that of *E. rothschildi* at OTU, phylum or genus level (Fig. S2). Firmicutes and Bacteroidota were dominant microbes in both of two species (Fig. 1). However, there are some differences in proportion between two species at phylum level. The proportion of Firmicutes was 69.97 and 51.31%, while Bacteroidota was 22.77 and 35.90% in *E. cansus* and *E. rothschildi*, respectively (Fig. 1). Firmicutes were more abundant in *E. cansus*, while the relative abundance of Bacteroidota, Desulfobacterota and Actinobacteriota were significantly higher in *E. rothschildi*. (Fig. 3). In addition, *E. cansus* harbored one unique Verrucomicrobia (< 1% relative abundance). The bacteria of this phylum were identified as genus Akkermansia.

**Fig. 2:**
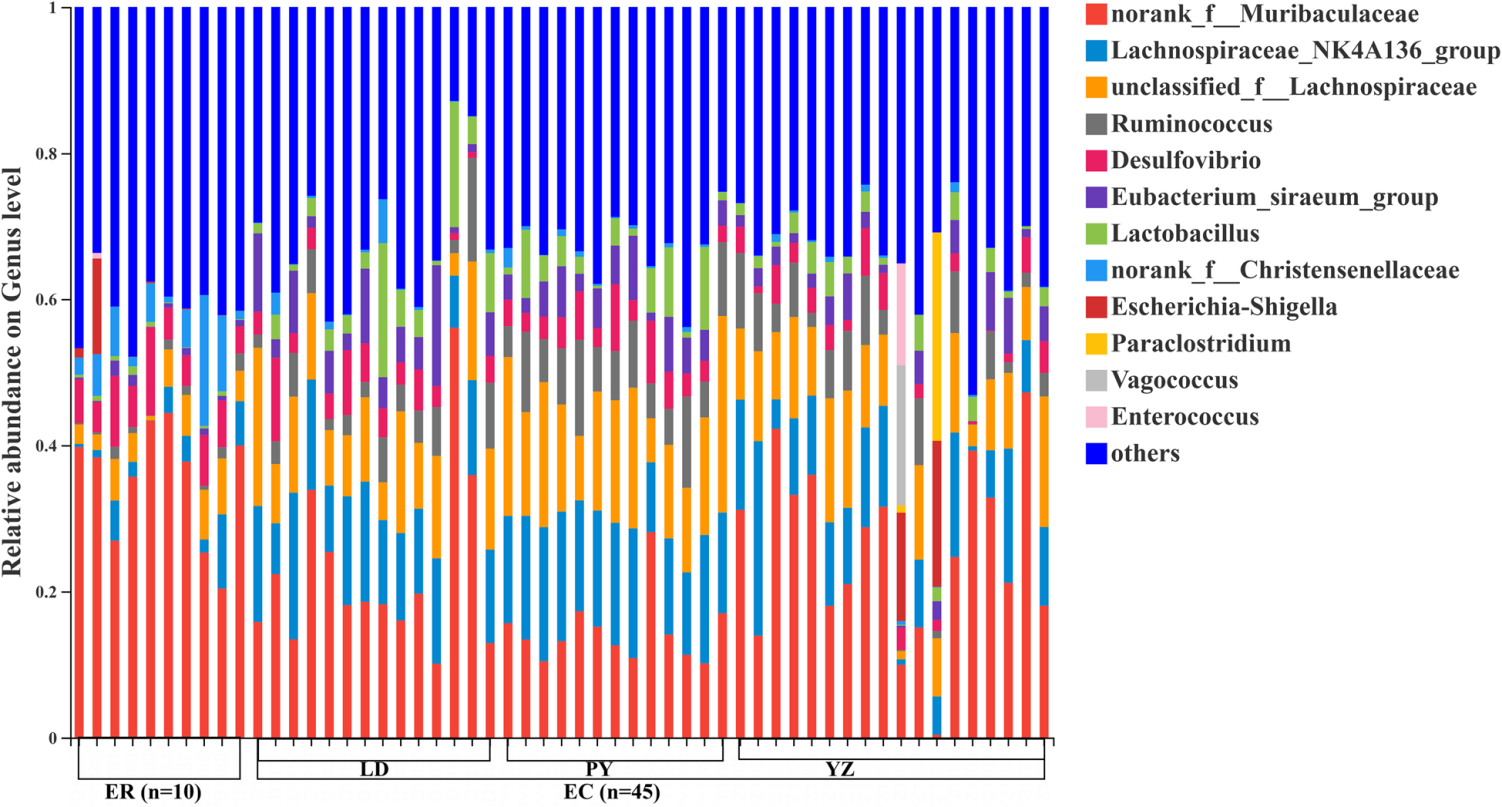
Mean relative abundances of bacteria at genus level across all zokor samples (EC, *E. cansus*; ER, *E. rothschildi*)

At genus level, *norank_f_Muribaculaceae* were dominant microbes in both of two species, and *unclassified_f_Lachnospiraceae*, *Lachnospiraceae_NK4A136_group*, *norank_f_Oscillospiraceae* and *Desulfovibrio* were also enriched in two zokor species (Fig. 2). The proportion of *norank_f_Muribaculaceae* was 21.59 and 35.20% in *E. cansus* and *E. rothschildi*, respectively (Fig. 2). There were 12 genera significantly different between two species (Fig. 4). *unclassified_f__Lachnospiraceae*, *Lachnospiraceae_NK4A136_group*, *Ruminococcus*, *Eubacterium_siraeum_group* and *Lactobacillus* were enriched in *E. cansus*, while *norank_f__Muribaculaceae*, *norank_f__Christensenellaceae*, *unclassified_f__Christensenellaceae*, *Oscillospiraceae*, *Desulfovibrio*, *norank_f_Oscillospiraceae* and *NK4A214_group* were more abundant in *E. rothschildi*. In addition, *E. cansus* harbored 36 unique genera, and the bacteria with relative abundance > 0.1% were *Paraclostridium*, *Vagococcus*, *Actinobacillus* and *unclassified_f__Prevotellaceae*. *E. rothschildi* harbored 11 unique genera, and the bacteria with relative abundance > 0.1% were *Treponema* and *unclassified_c__Bacteroidia*.

### Alpha diversity

Alpha diversity was estimated by four indices including Shannon, Chao, ACE and Simpson. We have found that there were no significant differences (*p* > 0.05) between two species on these four indices by Wilcoxon rank-sum test (Fig. S3).

We investigated whether trapping location, sex, and body measurements affected the alpha diversity values. There was a significant effect of trapping location on Chao and ACE indices within *E. cansus* (*p* < 0.05; Table 1). Samples from PY had significantly greater Chao (*p* < 0.01) and ACE (*p* < 0.05) indices than that of LD. However, there was no significant effect of trapping location on the Shannon and Simpson diversity. Besides, sex had no significant on alpha diversity of *E. cansus* (*p* > 0.05; Fig. S4). Sex had no significant on Shannon, Chao and ACE indices of *E. rothschildi* (*p* > 0.05; Fig. S5), however, males have greater Simpson index than that of females (*p* < 0.05).

**Table 1:** Mean alpha diversity (*±* SD) values for each trapping location. Letters after the values denote groups that differ significantly at *p* < 0.05 for alpha diversity

Trap site	Shannon	Chao	ACE	Simpson
LD	5.00 (0.48)	895.74 (67.61) a	880.19 (67.08) a	0.023514 (0.0217)
PY	5.30 (0.13)	971.43 (70.27) b	948.58 (65.66) b	0.014551 (0.0037)
YZ	4.96 (0.70)	925.99 (142.03) ab	908.44 (132.83) ab	0.026558 (0.0339)

The body measurements including weight, body length, weight/body length and tail length for each sample were shown in Table S1. We also searched for correlations (Pearson with two-tailed significance tests) between the alpha diversity and body measurements of *E. cansus* and *E. rothschildi*, respectively. We didn’t find the significant correlations between the values of alpha diversity and body measurements including weight, body length, weight/body length and tail length in both of two zokor species (*p* > 0.05; Table S2).

### Beta diversity

To intuitively demonstrate the differences of intestinal microbes between two species, principal coordinate analysis (PCoA) was performed based on Bray-Curtis and unweighted UniFrac distance to visualize the separation of gut microbiota structure across different species. By ranking the distance among samples, the order of samples in the low dimensional space can reflect the relationship among them. Principal coordinate analysis showed that the *E cansus* and *E. rothschildi* had distinct composition of intestinal microbes (Fig. 5). ANOSIM analysis confirmed that the structure of gut microbiota was significantly influenced by host species (Bray-Curtis, r = 0.649, *p* < 0.001; unweighted UniFrac, r = 0.742, *p* < 0.001), while sex had no significant effect on the gut microbiota (*p* > 0.05).

### Predicted metagenomes

The function of microbes of zokor based on COGs include carbohydrate transport and metabolism, transcription, amino acid transport and metabolism and other 21 functions (Table S3). The relative abundances of most of functions gene categories were extremely significantly higher in *E. cansus* than those in *E. rothschildi* (*p* < 0.01) except for extracellular structures (*p* > 0.05). Thereinto, carbohydrate transport and metabolism was the most important function.

## Discussion

### Intestinal microbes of zokor accord with the characteristics of herbivores

**Fig. 3:**
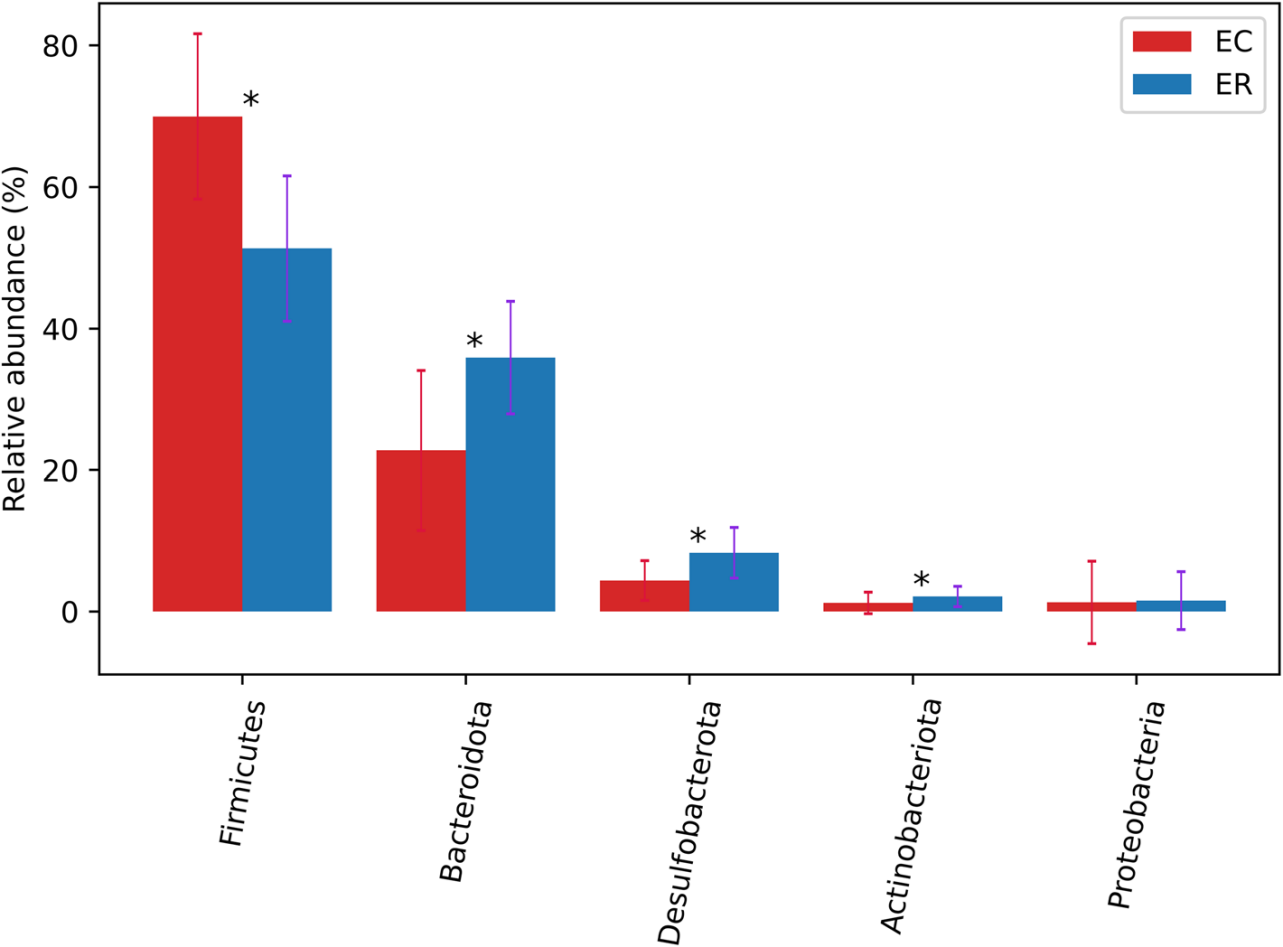
Differences of composition of intestinal microbes between two zokor species at phylum level (EC, *E. cansus*; ER, *E. rothschildi*). Significant difference is indicated by asterisk. *, *p* < 0.05; **, *p* < 0.01; ***, *p* < 0.001

**Fig. 4:**
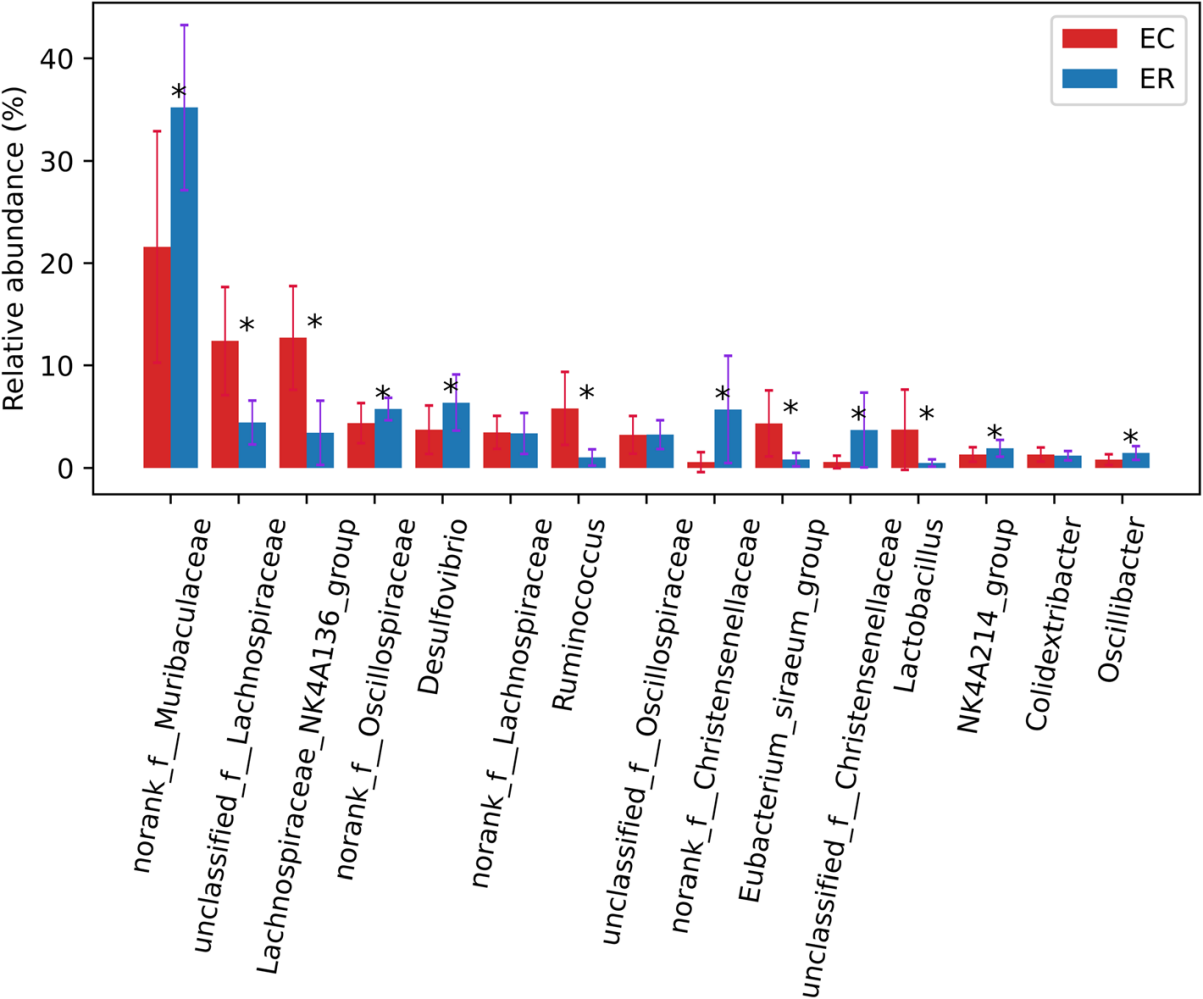
Differences of composition of intestinal microbes between two zokor species at genus level (EC, *E. cansus*; ER, *E. rothschildi*). Significant difference is indicated by asterisk. *, *p* < 0.05; **, *p* < 0.01; ***, *p* < 0.001

In this study, the composition and structure of intestinal microbes of *E. cansus* and *E. rothschildi* were studied by high-throughput sequencing technology. At phylum level, Firmicutes and Bacteroidota are dominant microbes in both of two zokor species. The total proportion of Firmicutes and Bacteroidota are 92.74 and 87.21% in *E. cansus* and *E. rothschildi*, respectively (Fig. 3). Firmicutes and Bacteroidota are mainly responsible for food fermentation in the gut [22]. High percentage of Firmicutes and Bacteroidota contributes to better decomposition of the cellulose and hemicellulose, and it accords with the characteristics of herbivores such as horse and donkey [23, 24]. At genus level, *norank_f_Muribaculaceae* are dominant genera in both of two species (Fig. 4). *Norank_f_Muribaculaceae* belong to the family S24–7, and S24–7 spp. are related to the degradation of a variety of complex carbohydrates [25]. Therefore, the dominant composition of intestinal microbes of *E. cansus* and *E. rothschildi* at phylum and genus level indicates that intestinal microbes of zokor are highly adaptive to phytophagous habits.

### Diversity of intestinal microbes and its correlation of two zokor species

**Fig. 5:**
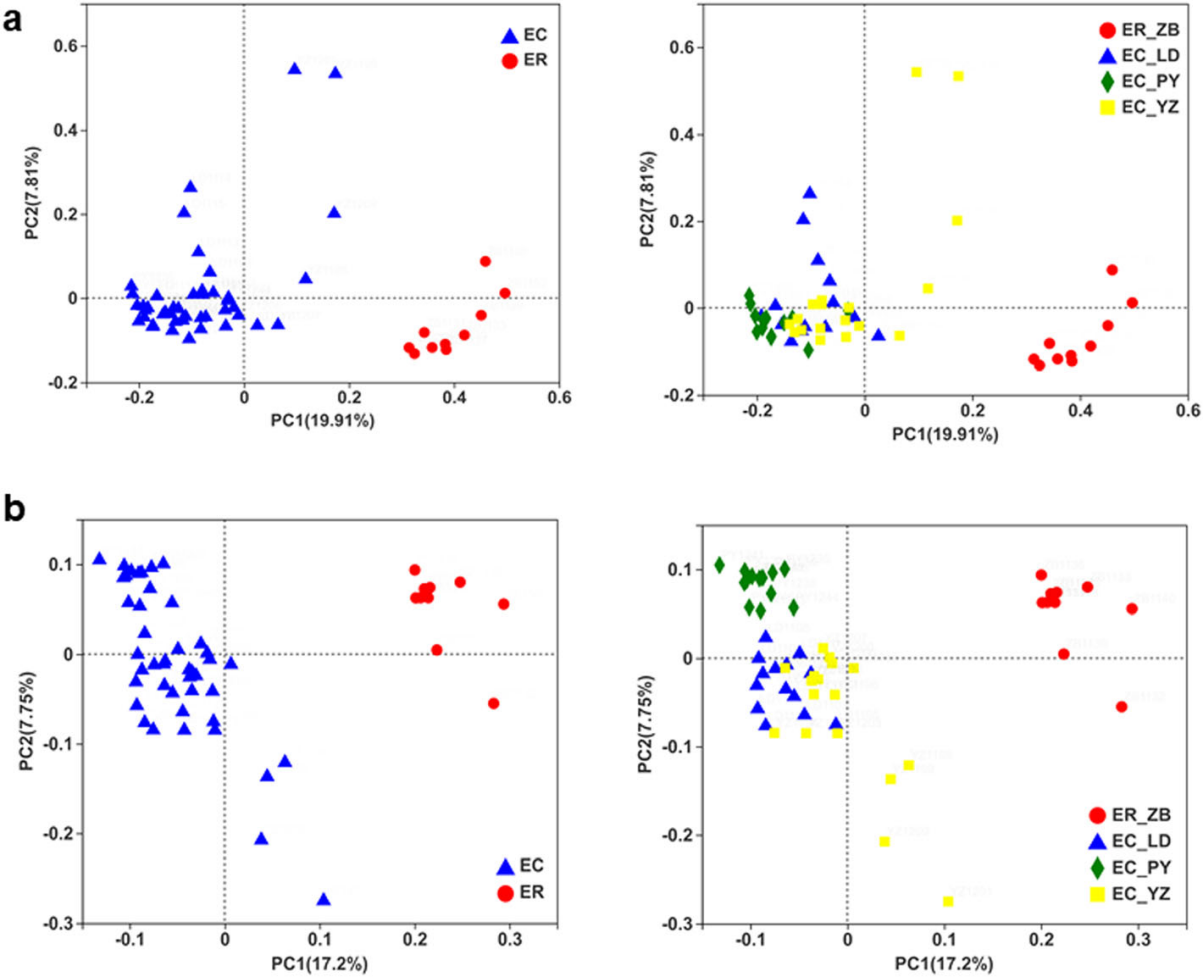
Principal coordinates analysis (PCoA) of bacterial communities of two zokor species across 55 samples. **a.** based on Bray-Curtis distance metrics (r = 0.649, *p* < 0.001). **b.** based on the unweighted UniFrac distance metrics (r = 0.742, *p* < 0.001). **Note:** EC, *E. cansus*; ER, *E. rothschildi*; ER_ZB: *E. rothschildi* in ZB; EC_LD: *E. cansus* in LD; EC_PY: *E. cansus* in PY; EC_YZ: *E. cansus* in YZ

Intestinal microbial diversity is an important indicator of stability and metabolic capacity [26, 27]. Intestinal microbes with high diversity provide a stronger capacity to utilize various metabolic pathways [28]. Although the composition of microbes of *E. cansus* were more abundant than that of *E. rothschildi* at OTU, phylum or genus level (Fig. S2), but it’s related to the larger sample size of *E. cansus*. Shannon, Chao, ACE and Shannon indices were with no significant differences between *E. cansus* and *E. rothschildi* (Fig. S3). It is suggested that the intestinal microbes of both two zokor species might be stable and have many functions. Beta diversity result (Fig. 5) showed that the samples of *E cansus* and the samples of *E. rothschildi* were clearly separated, demonstrated the structural divergence of intestinal microbes between two zokor species. Moreover, we have found that the composition and structure of intestinal microbes of *E. cansus* were more similar to each other than that of *E. rothschildi* regardless of geographical location, showing that the interspecific distance was larger than intraspecific distance. It is also suggested that the genetic background of host is an important factor in shaping intestinal microbes [29, 57]. Although two zokor species are closely related, the habitat types and diet resources were distinct between two zokor species. All of these factors may contribute to the divergence of intestinal bacteriome. Correspondingly, similar results have been found in pika [29] and ruminants [30].

To explore the influencing factor to alpha diversity, we found that there was a significant effect of trapping location on the alpha diversity (Table 1). *E. cansus* in LD had lower bacterial diversity than that of PY, which may be due to lower diet diversity of *E. cansus* resulted by lower diversity of plant communities in LD. In fact, we just observed significant differences on Chao and Ace indices. Chao and Ace indices [65, 66] could reflect the richness of bacterial communities while Shannon and Simpson [63, 64, 67] could reflect the diversity of bacterial communities. It is inferred that the diversity of bacterial species may not be easier to change than richness despite the far geographical distance between these two populations. We didn’t find significant effect of sex on alpha diversity of two zokor species (Fig. S4; Fig. S5). These results were consistent with precious study [16]. However, we only observed the differences of Simpson indices between two sexes in *E. rothschildi*, which may be resulted from small sample size of *E. rothschildi*. In addition. This was the first research on exploring the correlations between the alpha diversity and body measurements of zokor. We didn’t find the significant correlations between them (Table S2). However, significant correlations of microbiota diversity and body measurements including weight, body-mass index (weight/body length) were found in *Mus musculus* [31]. To make our results more reliable, more samples of zokors especially *E. rothschildi* should be collected and supplemented in further study.

### Comparison of intestinal microbes composition between two zokor species

Although the composition of intestinal microbes is similar in *E. cansus* and *E. rothschildi*, the proportion of bacterium are distinctly different between two species. The relative proportion of Firmicutes and Bacteroidota varied between the two zokor species (Fig. 3). *E. cansus* have a high proportion of Firmicutes/Bacteroidota than that of *E. rothschildi*. It is reported that high proportion of Firmicutes/Bacteroidota was related to carbohydrates diet [32], and was also related to obesity [33]. Firmicutes/Bacteroidota is usually used as an indicator to measure the status of obesity [34]. In the sampling process, we also found that the body size and fat thickness of *E. rothschildi* was significantly lesser than that of *E. cansus*. It is suggested that high proportion of Firmicutes/Bacteroidota of *E. cansus* contributes to obtaining more energy from dietary plants, especially complex carbohydrates. Verrucomicrobia were unique phylum in *E. cansus*. Species of Akkermansia (phylum Verrucomicrobia) are mucin-degrading bacteria, which may be capable of using mucin as carbon source for host [35]. Zokor especially *E. cansus* often faced with the lack of food resources due to its underground lifestyle, the Akkermansia within *E. cansus* may be beneficial for zokor to obtain energy from mucin.

At genus level (Fig. 4), the genera with significantly high proportion in *E. cansus* were related to the degradation of complex carbohydrates such as cellulose and hemicellulose. *Unclassified_f__Lachnospiraceae* and *Lachnospiraceae_NK4A136_group* both belong to Lachnospiraceae which are involved in metabolism as butyrate producer [36, 37]. And the latter was fibrolytic bacterium which can degrade the complex plant bran of recalcitrant substrate [38, 39]. Besides, species in *Ruminococcus* like *Ruminococcus albus* and *Ruminococcus flavefaciens* were widely studied in herbivores, and these two species have played a key role in decomposition of cellulose and hemicellulose [40–42]. In addition, *Eubacterium_siraeum_group* were proven to have the ability to ferment cellobiose to acetic acid [43], and *Lactobacillus* which have probiotic functions can ferment the carbohydrates to lactic acid [44]. It is indicated that intestinal microbes of *E. cansus* have stronger ability to ferment complex carbohydrates than that of *E. rothschildi*. Compared with *E. rothschildi* rich in edible plants, *E. cansus* just used limited resources as diet such as the roots of woody plants. These woody plants generally may have higher crude fiber than herbaceous plants [17]. Therefore, stronger ability of fermentation and degradation of cellulose of *E. cansus* is a long-time adaptation to limited food resources of its habitat. We have found significantly decreased *NK4A214_group* in *E. cansus* compared to *E. rothschildi*. Similar results have also been found in pigs, it is shown that the relative abundance of *NK4A214_group* were decreased when pigs were fed by wheat bran (fibrous) [45].

For *E. rothschildi*, the genera which were enriched in this species were related to the catabolism of various ingredient. *Norank_f_Muribaculaceae* might be related to the degradation of a variety of carbohydrates [25]. *Norank_f__Christensenellaceae* and *unclassified_f__Christensenellaceae* belong to the family Christensenellaceae, and species of this family has many physiological functions such as catabolism of protein and prebiotic fibers [46–49]. In addition, *Oscillospiraceae* and *norank_f_Oscillospiraceae* could utilize effectively carbohydrates and monosaccharide of tender leaves and roots [50]. Furthermore, species of *Desulfovibrio* were a group of sulphate-reducing bacterium which can decompose sulfate into hydrogen sulphide [51]. These bacteria enriched in *E. rothschildi* indicated the adaptation to the abundant food resources of its habitat.

Unique genera in *E. cansus* including *Paraclostridium*, *Vagococcus*, *Actinobacillus* and *unclassified_f__Prevotellaceae* were related to emerging pathogens [52–55]. Unique genus *Treponema* in *E. rothschildi* were also related to pathogens [56], however, the functions of *unclassified_c_Bacteroidia* were unknow. These unique genera of two zokor species may be host-specific bacteria, which may result from species loss and sorting due to the enrichment of nutrients [57].

PICRUSt metagenomic prediction showed that the relative abundances of the carbohydrate metabolism gene categories were significantly higher in *E. cansus* than those in *E. rothschildi* (Table S3). It is inferred that intestinal microbes of *E. cansus* would help hosts to maximize the nutrient utilization and energy extraction from indigestible plant carbohydrates, such as cellulose and hemicellulose.

This is the first time to report the composition and structure of intestinal microbes of *E. rothschildi*, however, the intestinal microbes of *E. cansus* was first reported in 2018 [16]. In this study, the intestinal microbes of *E. cansus* and *E. rothschildi* were investigated. It is shown that the abundance and diversity of intestinal microbes are distinct between two species, although there are little differences in composition of intestinal microbes. Furthermore, the intestinal microbes of these two species gradually differentiated in some functions especially the degradation of complex carbohydrates. These works would be significant to understanding the adaptive evolution of zokor and the process of species differentiation of Myospalacinae.

## Conclusions

In conclusion, our study demonstrates that the intestinal microbes between *Eospalax cansus* and *Eospalax rothschildi*. Are distinct, although there are little differences in composition of intestinal microbes. *Eospalax cansus* harbor a stronger ability of fermentation for dietary plants than that of *Eospalax rothschildi*. The stronger ability of fermentation and degradation of cellulose of intestinal microbes of *Eospalax cansus* may be a long-time adaptation to limited food resources underground.

## Methods

### Sample collection

Samples were collected from May to July in 2020. Wild adult *E. cansus* and *E. rothschildi* were captured from Ningxia Hui Autonomous Region and Shaanxi Province of China, respectively. The details of sampling information were shown in Table 2. After zokors were humanely euthanized by intravenous pentobarbital sodium (390 mg/mL) overdose after sedation with xylazine hydrochloride (5 mg/kg). The cecal contents were instantly collected from the ceca of wild zokors within 5 min of euthanasia, and immediately placed in cryogenic vials, frozen in liquid nitrogen, and then stored at − 80 °C in a refrigerator in the laboratory. 55 cecal samples were obtained from two species of zokors, including *E. cansus* (*n* = 45) and *E. rothschildi* (*n* = 10). All animal experiments were approved by the Institution of Animal Care. Processing of wild animals and sample collection were strictly congruent with the guidelines of our academic institution.

**Table 2:** Information of sampling area of zokor

Sampling locality	Species	Code	Longitude/°E	Latitude /°N	Altitude/m	Sample size (male/female)
Shenlin forest in Longde county	*E. cansus* (EC)	LD	105.9290	35.579	1824	14 (8/6)
Xiaoshigou village in Pengyang county	*E. cansus* (EC)	PY	106.8456	36.0273	1721	13 (3/10)
Hongzhuang forest in Yuanzhou	*E. cansus* (EC)	YZ	106.1143	35.8121	2202	18 (12/6)
Xingzi forest in Zhenba county	*E. rothschildi* (ER)	ZB	108.0207	32.5008	1624	10 (4/6)

The major plant community in each sampling site was identified based on morphological characteristics. Herbaceous plants including different families were dominated by *Rubus corchorifolius*, *Artemisia argyi*, *Plantago depressa*, *Viola diffusa* and *Rehmannia glutinosa* in the habitat of *E. rothschildi* in Zhenba county (ZB)*.* However, there were less plant species in the habitat of *E. cansus*, and woody plants were major composition. Economic forests are being developed over the land of *E. cansus* habitat in Longde county (LD). As a result, there were fewer weeds and lower diversity of plant communities. The plant community in LD were dominated by *Corylus heterophylla*, *Hemerocallis citrina* and *Isatis tinctorial*. *Malus pumila* and *Isatis tinctorial* were dominant plants in Pengyang county (PY), while *Larix gmelinii*, *Amygdalus davidiana*, *Hippophae rhamnoides* and *Urtica fissa* were dominant plants in Yuanzhou distinct (YZ). The phloem of roots even the entire root of these arbors and shrubs, and the grass roots and rhizomes of these herbaceous plants were used as the diets of zokors.

### DNA extraction

Total genomic DNA of zokor was extracted with a Stool Genome DNA Extraction Kit (Tiangen Inc.) from the cecal contents following the manufacturer’s protocol. DNA concentration and quality were determined using the Nanodrop 2000 Spectrophotometer (Thermo Scientific, Wilmington, USA). DNA were detected with 1% agarose gel extraction kit (Takara Inc.) and then purified and sequenced by Majorbio Bio-Pharm Technology Co. Ltd. (Shanghai, China).

### PCR amplification and MiSeq sequencing of 16S rRNA gene

The universal primer pair (338 F: 5′-ACTCCTACGGGAGGCAGCAG-3′, 806 R: 5′-GGACTACHVGGGTWTCTAAT − 3′) was used to amplify the 16S rRNA (V3-V4 hypervariable regions) by PCR system (GeneAmp 9700, ABI, USA) from cecal contents DNA [58]. The procedures of PCR amplification, with 25 μL reaction volume, including 1 μL DNA, 0.5 μL dNTPs mix (10 mM concentration of each dNTP), 5 × High GC Buffer 5 μL, 1.0 μL of each primer (10 μ mol**·**L^− 1^) and 0.25 μL Q5 high-fidelity DNA polymerase, with sterile distilled water added up to 25 μL volume. Amplification conditions were 3 min at 95 °C followed by 27 cycles of 95 °C for 30 s, 55 °C for 30s, 72 °C for 45 s, and a final extension at 72 °C for 10 min. PCR product were extracted from a 2% agarose gel and further purified using the AxyPrep DNA Gel Extraction Kit (Axygen Biosciences, Union City, CA, USA) and quantified using QuantiFluor™ -ST (Promega, USA) according to the manufacturer’s protocol.

Purified amplicons were pooled in equimolar and paired-end sequenced (2 × 300) on an Illumina MiSeq platform (Illumina, San Diego, USA) according to the standard protocols by Majorbio Bio-Pharm Technology Co. Ltd. (Shanghai, China).

### Processing of sequencing data

Raw fastq files were quality-filtered by Trimmomatic and merged by FLASH with the following criteria: (i) The reads were truncated at any site receiving an average quality core < 20 over a 50 bp sliding window. (ii) Sequences whose overlap being longer than 10 bp were merged according to their overlap with mismatch no more than 2 bp. (iii) Sequences of each sample were separated according to barcodes (exactly matching) and Primers (allowing 2 nucleotide mismatching), and reads containing ambiguous bases were removed.

Operational taxonomic units (OTUs) were clustered with 97% similarity cutoff using UPARSE (version 7.1 http://drive5.com/uparse/) with a novel ‘greedy’ algorithm that performs chimera filtering and OTU clustering simultaneously. The taxonomy of each 16S rRNA gene sequence was analyzed by RDP Classifier algorithm (http://rdp.cme.msu.edu/) against the Silva (SSU123) 16S rRNA database using confidence threshold of 70%.

### Bioinformatics analysis

QIIME Pipeline Version 1.9.1 [59] was used to analyze raw data. All reads were trimmed and then assigned to each sample based on their unique barcodes. After removing chimeras, all the reads were clustered into operational taxonomic units (OTUs) at a 97% sequence identity, and were identified at different level of classification [60, 61]. To standardize sampling efforts across samples, each sample was rarefied to the same number of reads (22,494 sequences). The rarefaction curves were generated based on the observed OTUs [62]. In addition, we calculated the shared and unique OTUs, phyla or genera between two zokor species. Shannon [63, 64], Chao [65], ACE [66] and Simpson [67] indices of gut microbiota were calculated by QIIME to evaluate the alpha diversity. We also investigated whether trapping location, sex, and body measurements affected the alpha diversity values. The body measurements including weight, body length, weight/body length and tail length were recorded for each sample. And we searched for correlations (Pearson with two-tailed significance tests) among the alpha diversity and body measurements. To assess beta diversity, principal coordinate analysis (PCoA) was performed based on Bray-Curtis and unweighted UniFrac distance to visualize the separation of gut microbiota structure across different species [68, 69].

### Statistical analysis

Statistical analyses were conducted through SPSS 23.0 software [70, 71]. The significant and the highly significant levels were 0.05 and 0.01, respectively. The differences between species were detected by Wlicoxon rank sum test [72].

### Predicted metagenomes

PICRUSTv1.1.0 [73] was used to predict the function based on the abundance of COGs. By comparing the 16S rRNA gene sequences with the reference genome database of microorganisms with known functions, the function can be predicted. Two-tailed t tests (Bonferroni corrected) were performed to test the differences of gene functions between *E. cansus* and *E. rothschildi*.

### Nucleotide sequence accession numbers

The raw data of 16S rRNA sequence were deposited into the NCBI Sequence Read Archive (SRA) database by accession number PRJNA664217 (http://www.ncbi.nlm.nih.gov/bioproject/664217).

## Supplementary Information

**Additional file 1 Table S1**. The values of body measurements for each zokor sample. **Table S2a**. Correlation coefficients (and *p* value) between alpha diversity values and a range of measures of E. cansus. Significant effects are shown as ** *p* < 0.01, * *p* < 0.05. **Table S2b**. Correlation coefficients (and p value) between alpha diversity values and a range of measures of *E. rothschildi*. Significant effects are shown as ** p < 0.01, * p < 0.05. **Table S3**. The function of microbes of zokor based on COGs.

**Additional file 2 Fig. S1** Rarefaction curves at OTU level (EC, *E. cansus*; ER, *E. rothschildi*). **Fig. S2** Venn diagram showing the overlap of intestinal microbes between two zokor species (EC, *E. cansus*; ER, *E. rothschildi*). **a.** at OTU level. **b.** at phylum level. **c.** at genus level. **Fig. S3** The comparisons of alpha diversity of intestinal microbes between two zokor species (EC, *E. cansus*; ER, *E. rothschildi*). **a.** Shannon diversity. **b.** Chao index. **c.** Ace index. **d.** Simpson diversity. Significant difference is indicated by asterisk. *, *p* < 0.05; **, *p* < 0.01; ***, *p* < 0.001. **Fig. S4** The comparisons of alpha diversity of intestinal microbes between males and females of *E. cansus*. Significant difference is indicated by asterisk. **Fig. S5** The comparisons of alpha diversity of intestinal microbes between males and females of *E. rothschildi*. Significant difference is indicated by asterisk.

## Data Availability

The original sequence data during the current study are available at the SRA by accession number PRJNA664217 (http://www.ncbi.nlm.nih.gov/bioproject/664217).
