# Supplementary figures and images for: Functional differentiation related to decomposing complex carbohydrates of intestinal microbes between two wild zokor species based on 16SrRNA sequences
Source: BMC Vet Res. 2021 Jun 11;17:216. doi: 10.1186/s12917-021-02911-z (PMC8196462; doi:10.1186/s12917-021-02911-z)

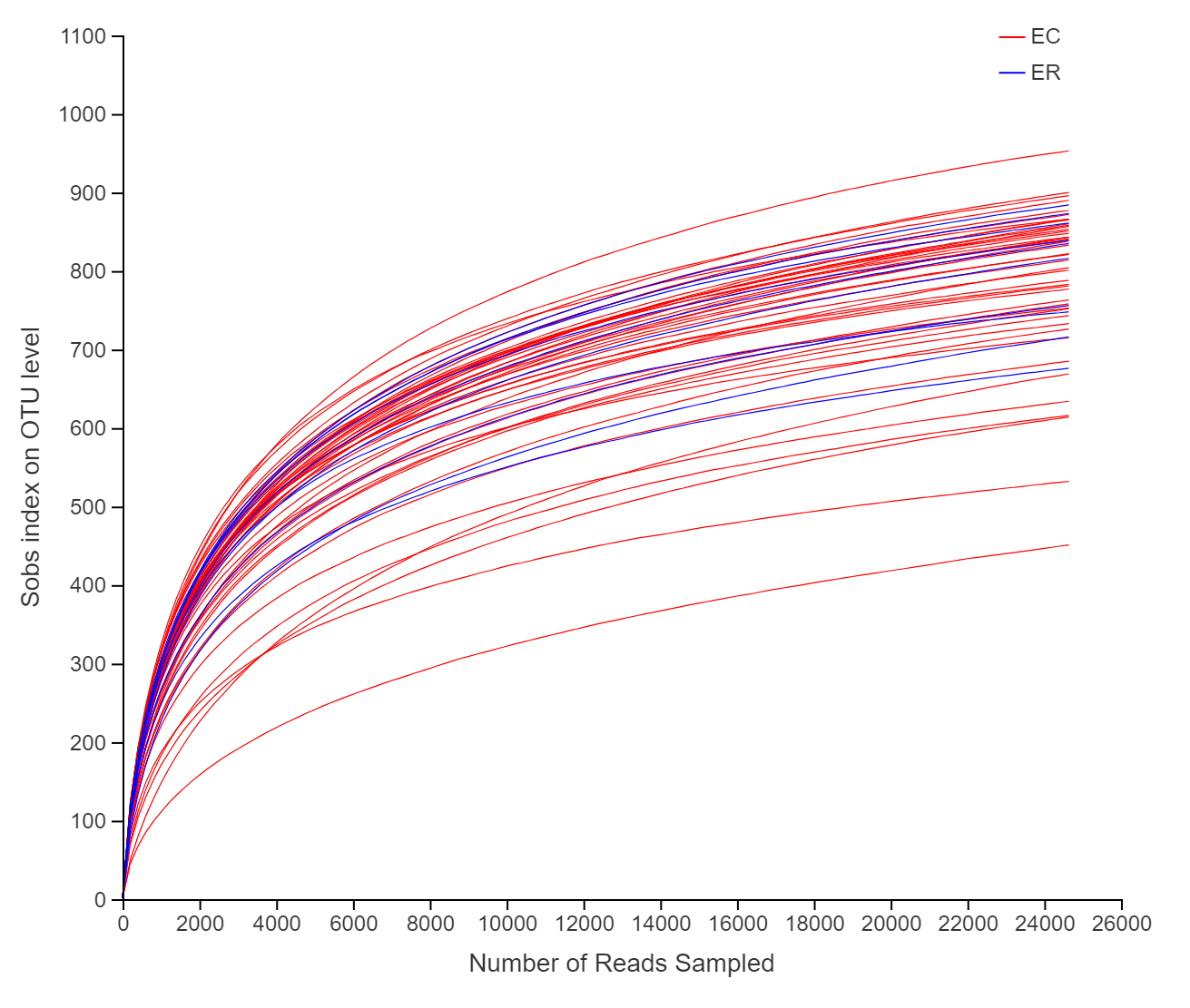

Supplement: Supplementary file 2 — Additional file 2 Fig. S1 Rarefaction curves at OTU level (EC, E. cansus; ER, E. rothschildi). Fig. S2 Venn diagram showing the overlap of intestinal microbes between two zokor species (EC, E. cansus; ER, E. rothschildi). a. at OTU level. b. at phylum level. c. at genus level. Fig. S3 The comparisons of alpha diversity of intestinal microbes between two zokor species (EC, E. cansus; ER, E. rothschildi). a. Shannon diversity. b. Chao index. c. Ace index. d. Simpson diversity. Significant difference is indicated by asterisk. *, p < 0.05; **, p < 0.01; ***, p < 0.001. Fig. S4 The comparisons of alpha diversity of intestinal microbes between males and females of E. cansus. Significant difference is indicated by asterisk. Fig. S5 The comparisons of alpha diversity of intestinal microbes between males and females of E. rothschildi. Significant difference is indicated by asterisk. [file 12917_2021_2911_MOESM2_ESM.zip › Fig. S1.png]

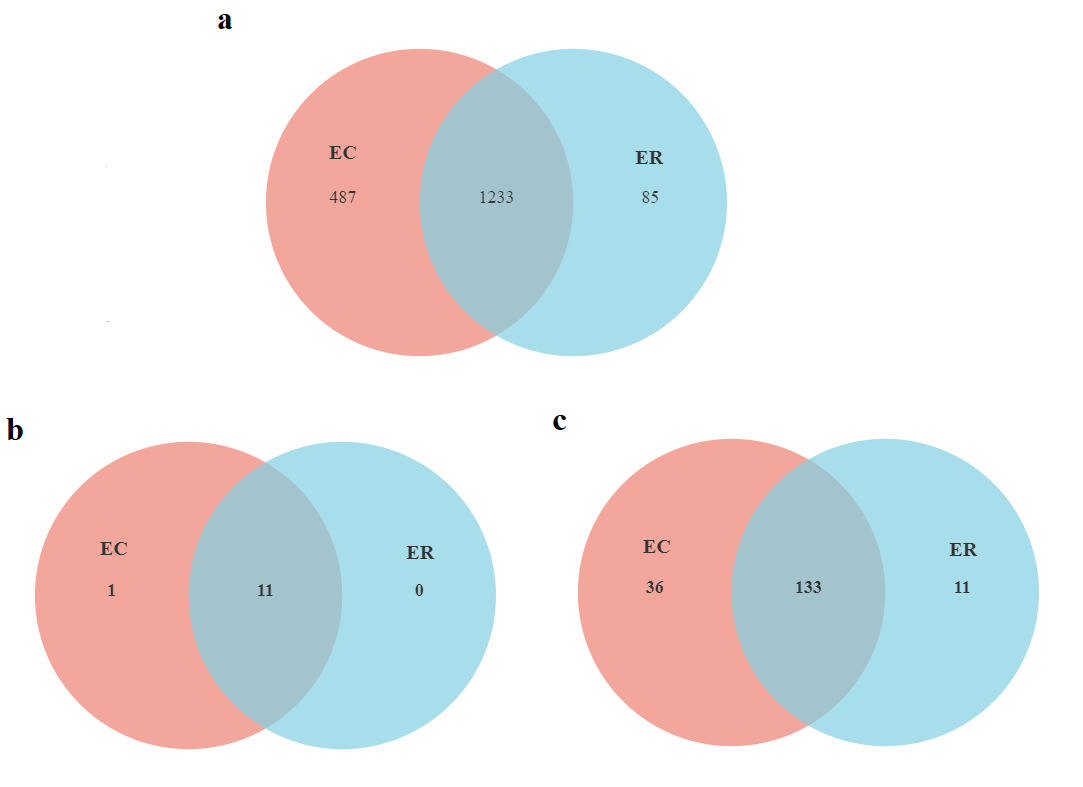

Supplement: Supplementary file 2 — Additional file 2 Fig. S1 Rarefaction curves at OTU level (EC, E. cansus; ER, E. rothschildi). Fig. S2 Venn diagram showing the overlap of intestinal microbes between two zokor species (EC, E. cansus; ER, E. rothschildi). a. at OTU level. b. at phylum level. c. at genus level. Fig. S3 The comparisons of alpha diversity of intestinal microbes between two zokor species (EC, E. cansus; ER, E. rothschildi). a. Shannon diversity. b. Chao index. c. Ace index. d. Simpson diversity. Significant difference is indicated by asterisk. *, p < 0.05; **, p < 0.01; ***, p < 0.001. Fig. S4 The comparisons of alpha diversity of intestinal microbes between males and females of E. cansus. Significant difference is indicated by asterisk. Fig. S5 The comparisons of alpha diversity of intestinal microbes between males and females of E. rothschildi. Significant difference is indicated by asterisk. [file 12917_2021_2911_MOESM2_ESM.zip › Fig. S2.png]

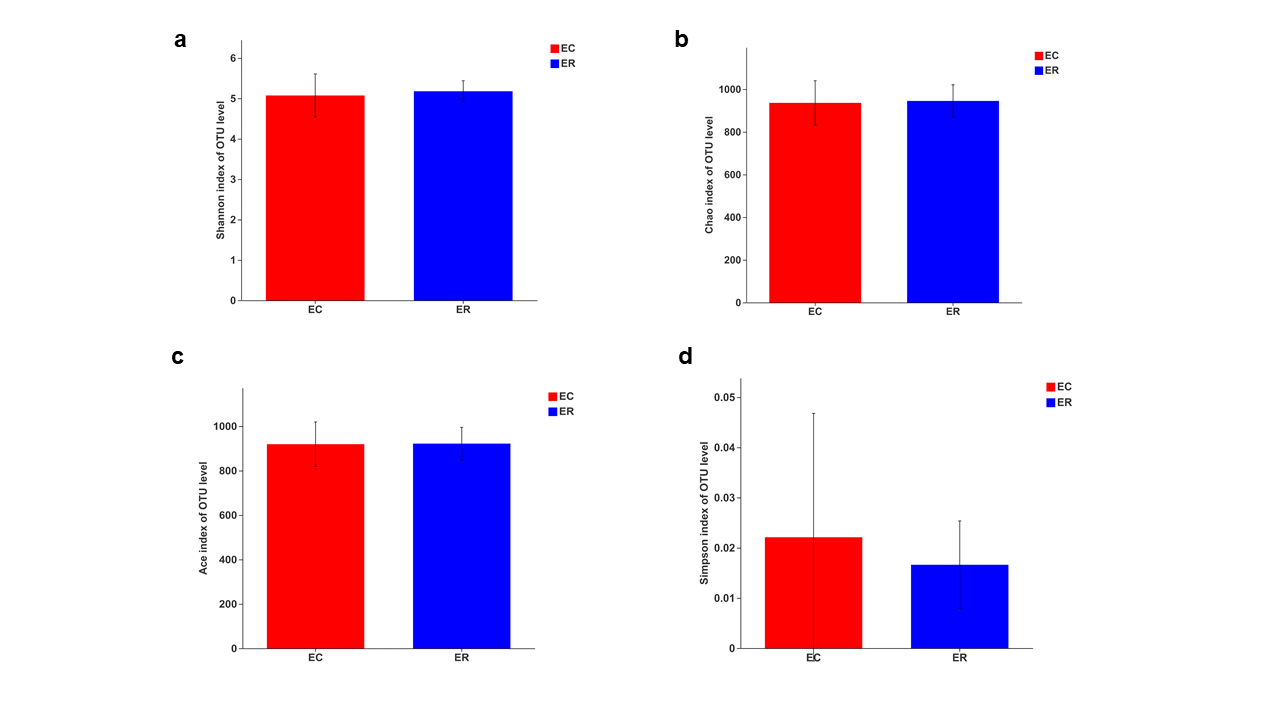

Supplement: Supplementary file 2 — Additional file 2 Fig. S1 Rarefaction curves at OTU level (EC, E. cansus; ER, E. rothschildi). Fig. S2 Venn diagram showing the overlap of intestinal microbes between two zokor species (EC, E. cansus; ER, E. rothschildi). a. at OTU level. b. at phylum level. c. at genus level. Fig. S3 The comparisons of alpha diversity of intestinal microbes between two zokor species (EC, E. cansus; ER, E. rothschildi). a. Shannon diversity. b. Chao index. c. Ace index. d. Simpson diversity. Significant difference is indicated by asterisk. *, p < 0.05; **, p < 0.01; ***, p < 0.001. Fig. S4 The comparisons of alpha diversity of intestinal microbes between males and females of E. cansus. Significant difference is indicated by asterisk. Fig. S5 The comparisons of alpha diversity of intestinal microbes between males and females of E. rothschildi. Significant difference is indicated by asterisk. [file 12917_2021_2911_MOESM2_ESM.zip › Fig. S3.png]

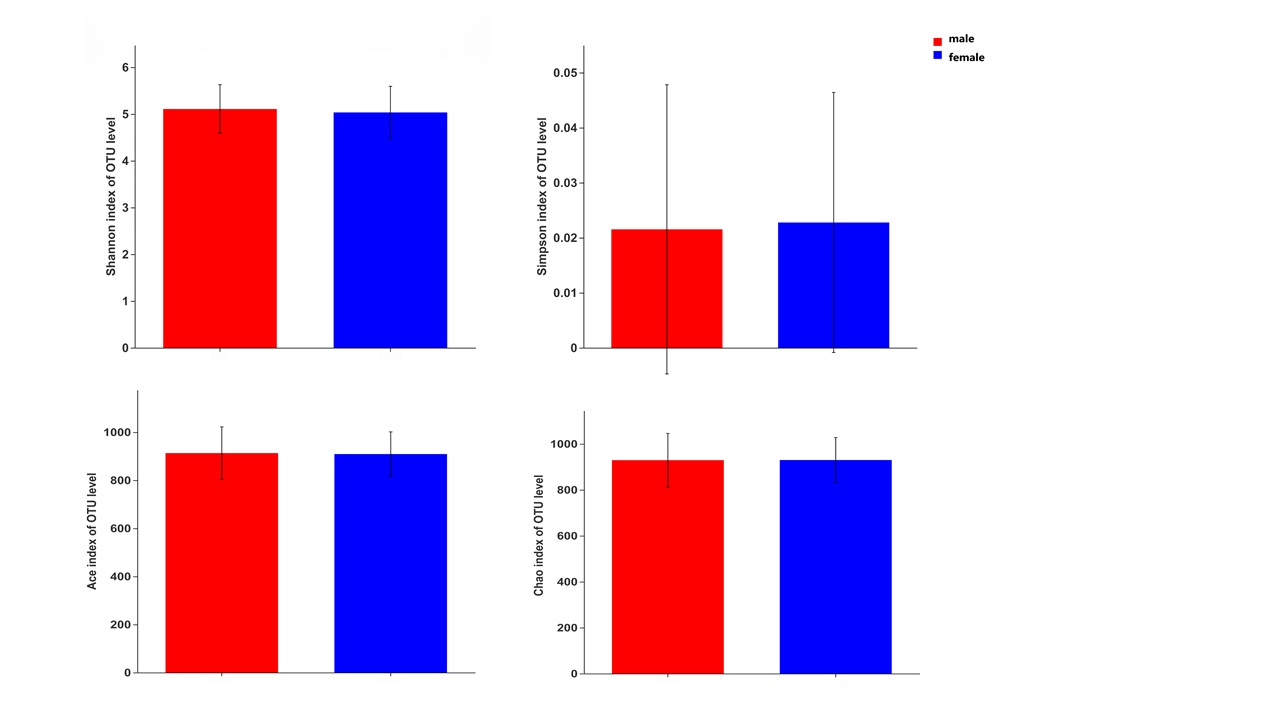

Supplement: Supplementary file 2 — Additional file 2 Fig. S1 Rarefaction curves at OTU level (EC, E. cansus; ER, E. rothschildi). Fig. S2 Venn diagram showing the overlap of intestinal microbes between two zokor species (EC, E. cansus; ER, E. rothschildi). a. at OTU level. b. at phylum level. c. at genus level. Fig. S3 The comparisons of alpha diversity of intestinal microbes between two zokor species (EC, E. cansus; ER, E. rothschildi). a. Shannon diversity. b. Chao index. c. Ace index. d. Simpson diversity. Significant difference is indicated by asterisk. *, p < 0.05; **, p < 0.01; ***, p < 0.001. Fig. S4 The comparisons of alpha diversity of intestinal microbes between males and females of E. cansus. Significant difference is indicated by asterisk. Fig. S5 The comparisons of alpha diversity of intestinal microbes between males and females of E. rothschildi. Significant difference is indicated by asterisk. [file 12917_2021_2911_MOESM2_ESM.zip › Fig. S4.png]

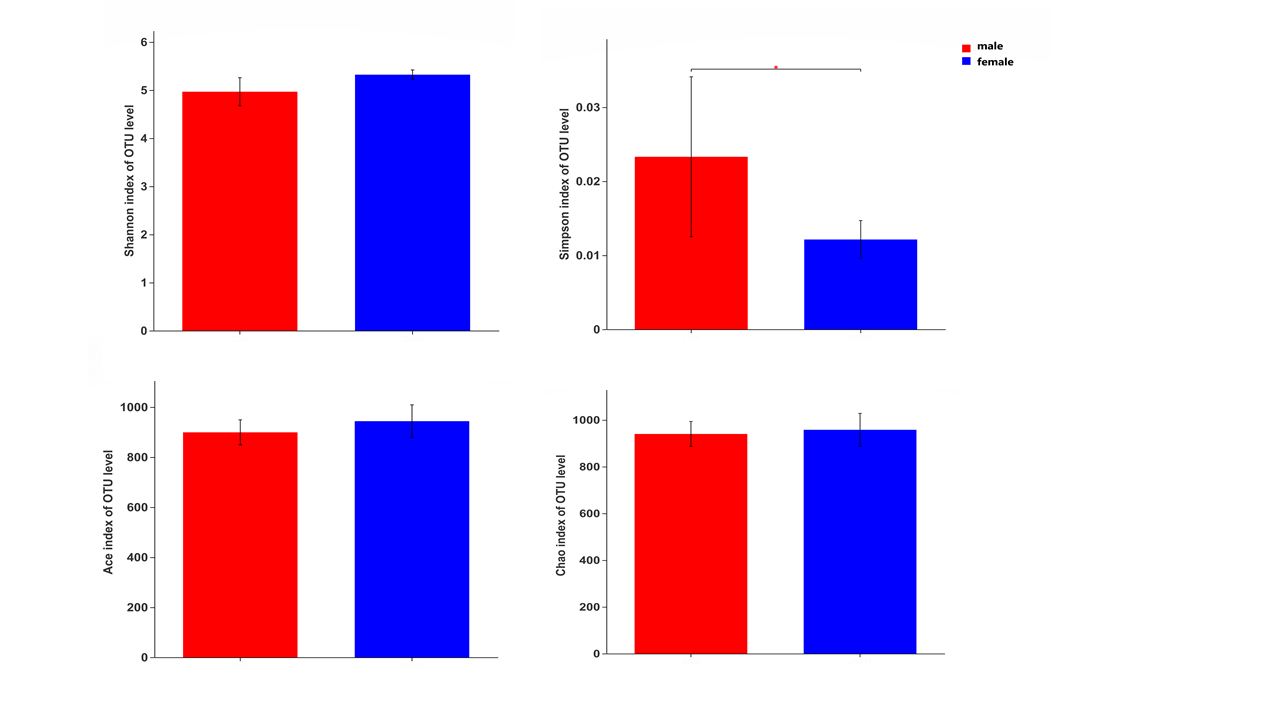

Supplement: Supplementary file 2 — Additional file 2 Fig. S1 Rarefaction curves at OTU level (EC, E. cansus; ER, E. rothschildi). Fig. S2 Venn diagram showing the overlap of intestinal microbes between two zokor species (EC, E. cansus; ER, E. rothschildi). a. at OTU level. b. at phylum level. c. at genus level. Fig. S3 The comparisons of alpha diversity of intestinal microbes between two zokor species (EC, E. cansus; ER, E. rothschildi). a. Shannon diversity. b. Chao index. c. Ace index. d. Simpson diversity. Significant difference is indicated by asterisk. *, p < 0.05; **, p < 0.01; ***, p < 0.001. Fig. S4 The comparisons of alpha diversity of intestinal microbes between males and females of E. cansus. Significant difference is indicated by asterisk. Fig. S5 The comparisons of alpha diversity of intestinal microbes between males and females of E. rothschildi. Significant difference is indicated by asterisk. [file 12917_2021_2911_MOESM2_ESM.zip › Fig. S5.png]
